# Analysis of dyadic coping patterns and influencing factors among patients with chronic heart failure and their primary caregivers based on latent profile analysis

**DOI:** 10.3389/fcvm.2026.1835727

**Published:** 2026-07-28

**Authors:** Chenming Li, Guyue Yan, Qi Zhang, Qing Wu, Yunying Hou

**Affiliations:** 1 Department of Cardiovascular Medicine, First Affiliated Hospital of Soochow University, Suzhou, Jiangsu, China; 2 School of Nursing, Medical College of Soochow University, Suzhou, Jiangsu, China

**Keywords:** chronic heart failure, dyadic coping, investigation and research, latent profile analysis, primary caregiver, quality of life

## Abstract

**Objective:**

To explore latent profiles of dyadic coping among patients with chronic heart failure and their primary caregivers, and to analyze the demographic characteristics and factors correlated with different profiles. This study aims to provide a reference for healthcare professionals in developing personalized dyadic coping intervention plans.

**Methods:**

A convenience sample of 320 dyads (patients with chronic heart failure and their primary caregivers) was recruited from the Department of Cardiovascular Medicine at a tertiary Grade A hospital in Suzhou between December 2024 and November 2025. Data were collected using questionnaires on general information, the Dyadic Coping Inventory (DCI), and the Self-Regulatory Fatigue Scale (SRF-S). Latent profile analysis (LPA) was employed to identify distinct profiles of dyadic coping within the dyads. Multinomial logistic regression was used to assess the influence of various factors on profile membership.

**Results:**

The mean dyadic coping scores were 125.28 ± 16.77 for patients and 126.08 ± 17.13 for caregivers. LPA identified four distinct latent profiles: Moderate Coping Harmony Group (46.25%), Low Coping Alienation Group (20.31%), Moderate-Low Coping Stress Group (19.38%) and High Coping Synergy Group (14.06%). Significant differences were found among the profiles regarding the patient's marital status, monthly household income per capita, NYHA functional class, and self-regulatory fatigue score, as well as the caregiver's gender, BMI, education level, caregiving duration, caregiving confidence, communication frequency with the patient, understanding of the patient's condition, and self-regulatory fatigue score (*P* < 0.05).

**Conclusion:**

Dyadic coping in chronic heart failure patient-caregiver dyads is heterogeneous. Healthcare providers should develop targeted interventions tailored to the specific profile characteristics and influencing factors, treating the patient and caregiver as an integrated unit to enhance dyadic coping for both parties.

## 1 Introduction

Chronic heart failure (CHF) represents a major global health challenge. Driven by factors such as accelerated population aging, advances in clinical diagnosis and treatment, and improved patient survival rates, the prevalence of CHF continues to rise (1). The Global Burden of Disease (GBD) study revealed approximately 56.19 million people worldwide suffered from CHF in 2019. The prevalence of adult heart failure (unstandardized prevalence) varies between 0.4% and 7% across different countries and regions (2). Chinese epidemiological data indicate that the total number of prevalent CHF cases rose from 7.03 million in 1990 to 18.51 million in 2019 (3). Multiple studies confirm that, beyond heart failure symptoms themselves, patients often have one or more non-cardiovascular comorbidities, further exacerbating adverse health outcomes such as increased mortality risk, reduced quality of life, and heightened healthcare resource utilization (4). This not only impacts patients' self-care capabilities and diminishes their quality of life, but also imposes a heavy caregiving burden on primary caregivers (5).

Dyadic coping refers to treating two individuals who maintain a significant sociological relationship as a single unit (6). Coping originates from stress theory, denoting an individual's response to resist stress events (7). In 1995, German scholar Bodenmann proposed the Systemic Interaction Model, emphasizing that spouses share an interdependent relationship during stress coping (8). Their responses to stressful events are not individual phenomena but a process of mutual interaction. Bodenmann defined the shared responses and strategies of spouses facing dyadic stressors as Dyadic Coping. The process of mutual support and joint coping with the same stressor constitutes the establishment of a dyadic relationship. Effective dyadic coping promotes physical and mental health, reduces negative emotions, thereby enhancing both partners' quality of life and improving patient recovery outcomes (9).

In recent years, dyadic coping research worldwide has become increasingly systematic and diversified, extending beyond marital contexts. Studies indicate that parental illness constitutes a stressor for both parents and children, and dyadic coping can enhance disease management capabilities and family quality of life (10). Latent profile analysis treats patients and their primary caregivers as a single analytical unit, categorizing them into distinct performance subgroups to explore dyadic coping patterns. This approach has been applied in studies on cancer (11), pregnancy complications (12), and stroke (13). However, dyadic coping patterns in chronic heart failure patients and their primary caregivers remain unexplored. This study will employ latent profile analysis to investigate dyadic latent profiles among heart failure patients and their primary caregivers, analyze influencing factors, and provide reference for healthcare professionals in designing and implementing personalized dyadic coping intervention programs.

## 2 Methods

### 2.1 Study population

This study employed convenience sampling to recruit CHF patients hospitalized in the Department of Cardiology at the First Affiliated Hospital of Soochow University from December 2024 to November 2025, along with their primary caregivers.

#### 2.1.1 Inclusion criteria

Patients with Chronic Heart Failure: Patients diagnosed with chronic heart failure were required to meet the diagnostic criteria specified in the “Chinese Guidelines for Diagnosis and Treatment of Heart Failure 2024" (14) and receive a confirmed clinical diagnosis of chronic heart failure. All eligible patients were aged 18 years or older, capable of completing questionnaires via written or verbal communication, and provided signed informed consent for voluntary participation in this study.

Primary Caregivers: Primary caregivers were required to be at least 18 years of age with intact communication ability. They needed to provide no less than four hours of daily care for the patients and continue to act as the primary caregiver after hospital discharge (15). All eligible caregivers should have sound communication and comprehension capabilities, be able to fully understand the questionnaire content, and participate in this study on a voluntary basis.

#### 2.1.2 Exclusion criteria

Patients with chronic heart failure: Patients were excluded if they suffered from severe physical diseases including cancer and major organ failure, had psychological disorders or a history of mental illness, or had received heart transplantation. In addition, individuals who had recently encountered major life events (e.g., family bereavement and accidental injury) were also excluded from this study.

Primary caregivers: Primary caregivers were excluded if they suffered from severe mental or psychological disorders, received payment for providing care, or had severe impairments in intelligence, hearing, vision or verbal communication.

#### 2.1.3 Sample size

Based on unordered multinomial logistic regression analysis, the sample size should be at least 10–15 times the number of independent variables^17^. This study included 25 independent variables. Considering a 10% non-response rate, the required sample size ranged from 278 to 417 pairs. The study planned to enroll 330 patient-primary caregiver dyads (approved by the Ethics Committee of Soochow University, approval number: SUDA2025010; China clinical trial registration number: ChiCTR2600117953).

### 2.2 Research tools

#### 2.2.1 General information questionnaire

Designed by the researchers after reviewing relevant literature, including a patient general information questionnaire (demographic and disease-related data) and a primary caregiver general information questionnaire. Patient demographic data: gender, age, body mass index (BMI), marital status, education level, per capita monthly household income, medical expense payment method, primary caregiver relationship, etc. Patient disease-related data: Duration of illness, New York Heart Association Functional Classification (NYHA), number of comorbidities, N-terminal pro-brain natriuretic peptide (NT-pro BNP), left ventricular ejection fraction (LVEF), etc. The clinical data were collected by investigators through the hospital electronic medical record system.

Primary caregiver general information questionnaire: Gender, age, BMI, educational attainment, physical condition, duration of caregiving, average daily caregiving hours, confidence in caring for the patient, communication with the patient, understanding of the patient's condition, etc.

#### 2.2.2 Dyadic coping scale

The Dyadic Coping Scale was developed by Bodenmann and adapted into Chinese by Xu (16, 17). It comprises 37 items across six dimensions: stress communication, supportive coping, vicarious coping, collaborative coping, negative coping, and coping quality evaluation. The scale exhibits a Cronbach's *α* coefficient of 0.91. All items use a 5-point Likert scale, where 1 indicates “not at all” and 5 indicates “extremely.” Higher scores indicate greater levels of dyadic coping within that dimension. Calculation method: negative coping items are reversely scored. The total scale score is derived from the sum of scores across all dimensions, with a theoretical range from 35 to 175. Scale scores are positively correlated with dyadic coping ability, meaning higher scores indicate a higher level of dyadic coping. Specific grading criteria are as follows: <111 points indicate low dyadic coping, 111–145 points indicate moderate dyadic coping, and >145 points indicate high dyadic coping. Based on the System Interaction Model (8), this study measured both patients and their primary caregivers separately. To accurately assess dyadic coping, each “patient-primary caregiver” pair completed the scale independently in non-interfering environments. This approach is widely adopted in studies involving chronic disease patients and primary caregivers (18).

It should be noted that the original Dyadic Coping Inventory (DCI) and its Chinese version were initially developed specifically for couples and underwent validity and reliability testing. In this study, non-spousal patient-care pairs—including children, parents, siblings, etc.—accounted for 28.7% of the total sample. To demonstrate the applicability of the DCI to this mixed familial sample, two key arguments are presented: First, Bodenmann's systematic interaction dyadicmodel does not restrict dyadicunits to couples; any pair sharing chronic disease stress and mutual dependence (e.g., patients with adult children or patients with parents) can constitute a complete dyadicsystem. Second, extensive empirical research applying the DCI to non-spousal chronic disease care pairs has confirmed that all six core dimensions of the scale effectively reflect interpersonal stress communication and collaborative coping patterns, independent of familial relationship type (19).Meanwhile, the group reliability analysis based on this study's data further confirms the scale's stability: the Cronbach's *α* coefficient was 0.908 for the spouse group and 0.892 for the non-spouse group, both demonstrating excellent internal consistency and confirming the scale's consistent psychometric performance across different family pairing samples.

#### 2.2.3 Self-regulation fatigue scale

The Self-Regulation Fatigue Scale was developed by Nes in 2013 and adapted into Chinese by Wang in 2015 (20, 21). It comprises 16 items across three dimensions: cognitive control, emotional control, and behavioral control. The scale employs a 5-point Likert scale, with each item scored from 1 (strongly disagree) to 5 (strongly agree). Higher scores indicate greater self-regulation fatigue. The Cronbach's *α* coefficient for this scale is 0.84. In practical application, this study follows the field's paradigm of measuring both parties' perceptions, administering the scale separately to patients and their primary caregivers. For instance, Chai Xiaopei (22) employed this approach to effectively measure self-regulation fatigue levels in both patients and caregivers, supporting the tool's applicability in dyadic research. This study adopts the same methodology to independently collect data from both parties, facilitating subsequent paired analysis and comparison.

### 2.3 Data collection

Data were collected via questionnaire one day prior to patient discharge. The questionnaires were administered by two trained graduate nursing students. Prior to the survey, the research objectives, significance, and instructions for completing the questionnaire were explained to patients and their primary caregivers. Questionnaires were distributed only after obtaining informed consent from both parties. All questionnaires were completed on-site, collected immediately, and verified. Any unclear items were promptly clarified. A total of 330 questionnaires were distributed and collected from heart failure patients and their primary caregivers. Ten sets of questionnaires with inconsistent or duplicate responses were excluded, leaving 320 valid sets.

### 2.4 Statistical analysis

Data were analyzed using Mplus 8.3 and SPSS 26.0. Dyadic coping responses from chronic heart failure patients and their primary caregivers on each dimension item were treated as manifest variables. Latent profile analysis was conducted in Mplus 8.3 to identify distinct patterns of dyadic coping profiles. The analysis employed a “stepwise increase in category number” approach: starting from a single-category model, multi-category models were sequentially constructed. The optimal model fit was determined based on model fit indices and clinical relevance. Model fit indices and their significance: Likelihood Ratio Chi-Square G2 (LL) test, Akaike information criteria (AIC), Bayesian information criteria (BIC), and adjusted BIC (aBIC). Lower values indicate better model fit. Entropy ranges from 0 to 1, with values closer to 1 indicating more precise classification. Likelihood ratio tests (Lo-Mendell-Rubin, LMR) and Bootstrapped likelihood ratio tests (BLRT) were used for model comparison. A significant test result (*P* < 0.05) indicates that model K fits better than model K-1. Based on the Systemic Interaction Model (8), this study regards patients and their primary caregivers as an interdependent unit. In the variable selection process, the factors that may affect the quality of interaction between the two parties were given particular attention, including the demographic and sociological data of the patients, the disease-related data of the patients, and the general questionnaire of the primary caregivers. Univariate analysis was performed for intergroup comparisons of age, sex, and other variables across latent profiles in heart failure patients and their primary caregivers. Chi-square *χ^2^* tests or Fisher's exact probability tests were used to assess distribution differences in dyadic coping across latent profiles. Multinomial logistic regression analysis was employed to investigate factors influencing dyadic coping in heart failure patients and their primary caregivers. Statistical significance was set at *P* < 0.05 or *P* < 0.01.

### 2.5 Technical workflow

**Figure 1:**
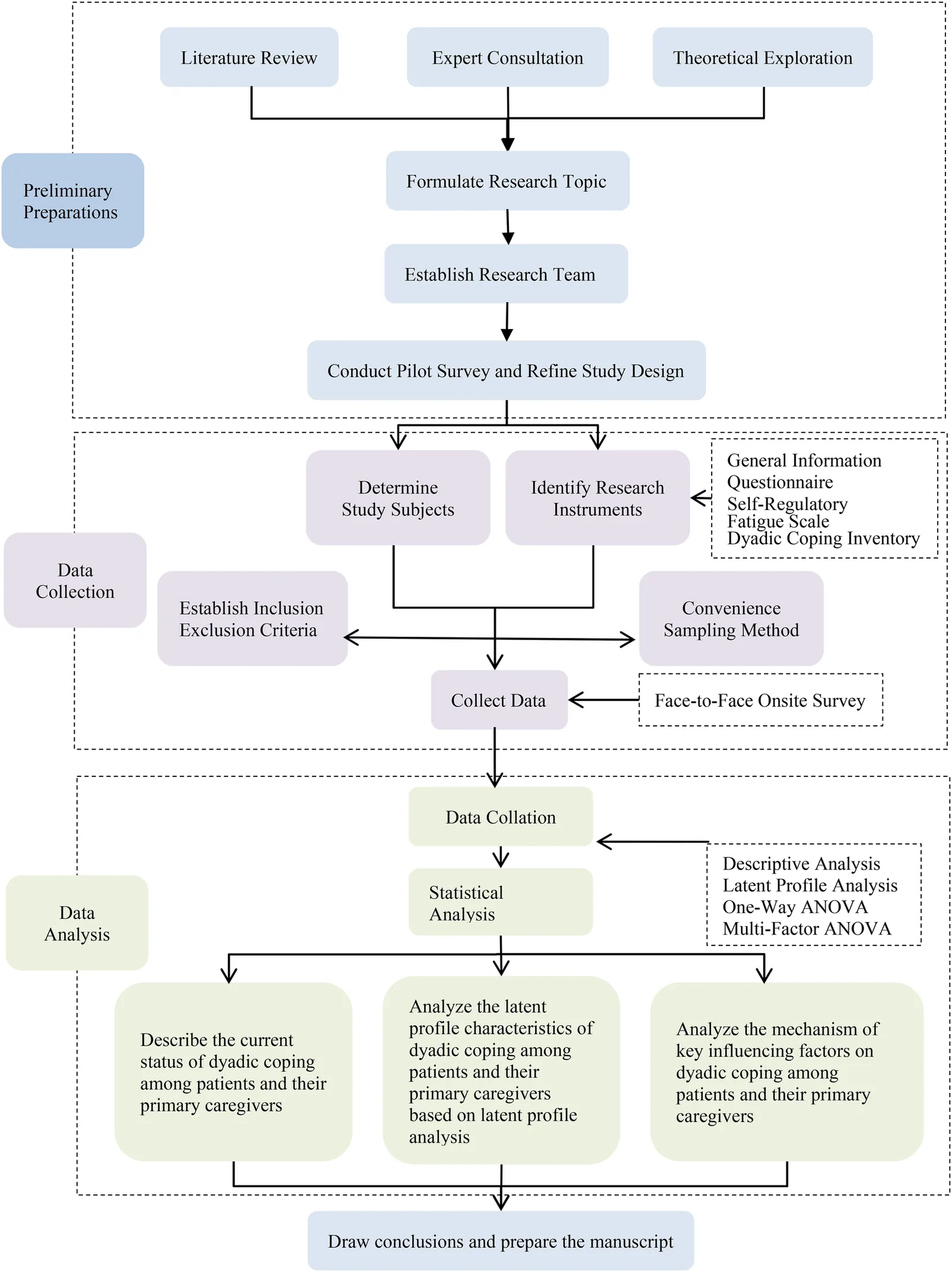
Technical workflow.

This study followed a standardized technical workflow. First, researchers communicated with heart failure patients and their primary caregivers and screened participants strictly based on the established inclusion and exclusion criteria. Second, trained research staff distributed three questionnaires to eligible dyads one day prior to patient discharge, provided on-site guidance for questionnaire completion, and collected all valid responses immediately. Third, the data were sorted, coded and imported into Mplus 8.3 and SPSS 26.0 for statistical analysis. Latent profile analysis was used to identify different dyadic coping profiles, and univariate analysis and multinomial logistic regression were further conducted to explore influencing factors. Finally, we summarized the findings, discussed relevant mechanisms, and put forward targeted clinical intervention recommendations (Figure 1).

## 3 Results

### 3.1 General characteristics of heart failure patients and their primary caregivers

A total of 330 dyads were initially recruited, and 320 valid questionnaires were finally included, with an effective response rate of 96.97%. Among 320 pairs of heart failure patients and their primary caregivers, patient ages ranged from 20 to 87 years, with a mean age of (66.02 ± 12.44) years. Males accounted for 211 cases (65.90%). Educational attainment was primarily elementary school or below (40.30%), with 164 (51.20%) having a household monthly income of ¥3,000–5,000. Duration of illness ranged from 0 to 2 years (63.70%), NYHA class III was present in 164 (51.20%), and 185 (57.80%) had three or more comorbidities. Primary caregivers ranged in age from 25 to 87 years, with a mean age of (59.11 ± 13.88) years. Among them, 199 (62.20%) were female, 97 cases (30.30%) had a junior high school education, 159 (49.70%) reported fair physical health, 200 (62.50%) frequently communicated with patients, and 215 (67.20%) had some understanding of patients' conditions (Tables 1, 2).

**Table 1:** General characteristics of patients (*n*=320).

Item	Category	*χ¯ **±s/n*****(%)** ***/M*****(*****p*****25 ,** ***p*****75)**
Age (years)		66.02 ± 12.44
Gender	Male	211 (65.90)
	Female	109 (34.10)
BMI (kg/m^2^)		23.85 ± 3.82
Marital Status	Married	271 (84.70)
	Unmarried	5 (1.60)
	Divorced	6 (1.90)
	Widowed	38 (11.90)
Educational Attainment	Elementary school or below	129 (40.30)
	Junior High	97 (30.30)
	High School or Technical School	64 (20.00)
	College or above	30 (9.40)
Monthly Per Capita Income (CNY)	＜3,000	70 (21.90)
	3,000–5,000	164 (51.20)
	>5,000	86 (26.90)
Payment Method	Self-funded	1 (0.30)
	Medical Insurance	274 (85.60)
	Rural Insurance	45 (14.10)
Primary Caregiver	Parents	9 (2.80)
	Spouse	228 (71.30)
	Children	81 (25.30)
	Siblings	2 (0.60)
Duration of Illness (Years)	0–2	204 (63.70)
	3–5	89 (27.80)
	6–8	15 (4.70)
	≥9	12 (3.80)
NYHA	II	104 (32.50)
	III	164 (51.20)
	IV	52 (16.30)
Number of Comorbidities (units)	0	10 (3.10)
	1	41 (12.80)
	2	84 (26.30)
	≥3	185 (57.80)
NT-proBNP(pg/mL)		2,542.56 (469.00,2,945.00)
LVEF(%)		39.36 (29.00,50.75)

BMI, body mass index; NYHA, New York Heart Association functional classification; NT-proBNP, N-terminal B-type natriuretic peptide precursor; LVEF, Left ventricular ejection fraction.

**Table 2:** General characteristics of primary caregivers (*n* = 320).

Item	Category	*¯χ **±s/n*****(%)** ***/M*****(*****p*****25 ,** ***p*****75)**
Age (years)		59.11 ± 13.88
Gender	Male	121 (37.81)
	Female	199 (62.29)
BMI (kg/m^2^)		23.60 ± 2.26
Education Level	Elementary school or below	94 (29.40)
	Junior High	100 (31.30)
	High School or Technical School	60 (18.80)
	College and above	66 (20.60)
Physical condition	Poor	31 (9.70)
	Average	159 (49.70)
	Healthy	130 (40.60)
Years of Caregiving	0–2	225 (70.30)
	3–5	74 (23.10)
	6–8	11 (3.40)
	≥9	10 (3.10)
Daily Care Duration (hours/day)	<5	168 (52.50)
	5–8	136 (42.50)
	>8	16 (5.00)
Care Confidence	Yes	239 (74.7)
	No	81 (25.30)
Communication	Frequently	200 (62.50)
	Occasionally	120 (37.50)
Understanding of condition	No knowledge	3 (0.9)
	Some understanding	215 (67.20)
	Very familiar	102 (31.90)

### 3.2 Latent profile analysis of dyadic coping among heart failure patients and their primary caregivers

#### 3.2.1 Current status of dyadic coping among patients and primary caregivers

The dyadic coping scores for heart failure patients and their primary caregivers were 84.00–169.00 (125.28 ± 16.77) points and 79.00–173.00 (126.08 ± 17.13) points, respectively. The difference in total dyadic coping scores between the two groups was statistically significant (*t* = −2.2, *P* < 0.05).

#### 3.2.2 Latent profile analysis of dyadic coping in patients and primary caregivers

Using the total scores of dyadic coping responses from heart failure patients and their primary caregivers as indicator variables, latent profile analysis was conducted to identify distinct patient-caregiver dyadic coping response patterns. The optimal model was determined to be the 4-profile model. The rationale is as follows (1):Although AIC, BIC and aBIC values continued to decline when expanding the number of profiles from four to five, the reduction magnitude was marginal, meaning the five-profile model brought only trivial improvements to model fit (2);The five-profile model suffered severe sample imbalance: the smallest subgroup accounted for merely 2.2% of all dyads (approximately seven pairs). Such a tiny sample size cannot support stable multinomial logistic regression analysis and lacks clinical representativeness (3); The fifth subgroup had no independent clinical value. It only split one original four-profile subgroup into two highly overlapping clusters with no unique dyadic coping characteristics, and this extra subdivision offered no reference for clinical intervention (4);The entropy value of the four-profile model reached 0.871 (≥ 0.8), reflecting accurate classification and unambiguous subgroup allocation. The LMR test (*P* = 0.0422) and BLRT test (*P* < 0.001) both yielded statistically significant results. Accordingly, all subsequent analyses were carried out based on the four latent profiles (Table 3).

**Table 3:** Model fit indices for latent profile analysis of dyadic coping in heart failure patients and their primary caregivers.

Category	AIC	BIC	aBIC	pLMR	pBLRT	Entropy	Probability of each category
1	5,444.917	5,459.991	5,447.303				1
2	5,113.150	5,139.528	5,117.325	<0.001	<0.001	0.902	0.381/0.619
3	5,019.241	5,056.924	5,025.206	0.0273	<0.001	0.852	0.350/0.503/0.147
4	4,930.806	4,979.794	4,938.560	0.0422	<0.001	0.871	0.194/0.141/0.203/0.462
5	4,867.570	4,927.863	4,877.114	0.0169	<0.001	0.895	0.203/0.191/0.409/0.175/ 0.022

**Figure 2:**
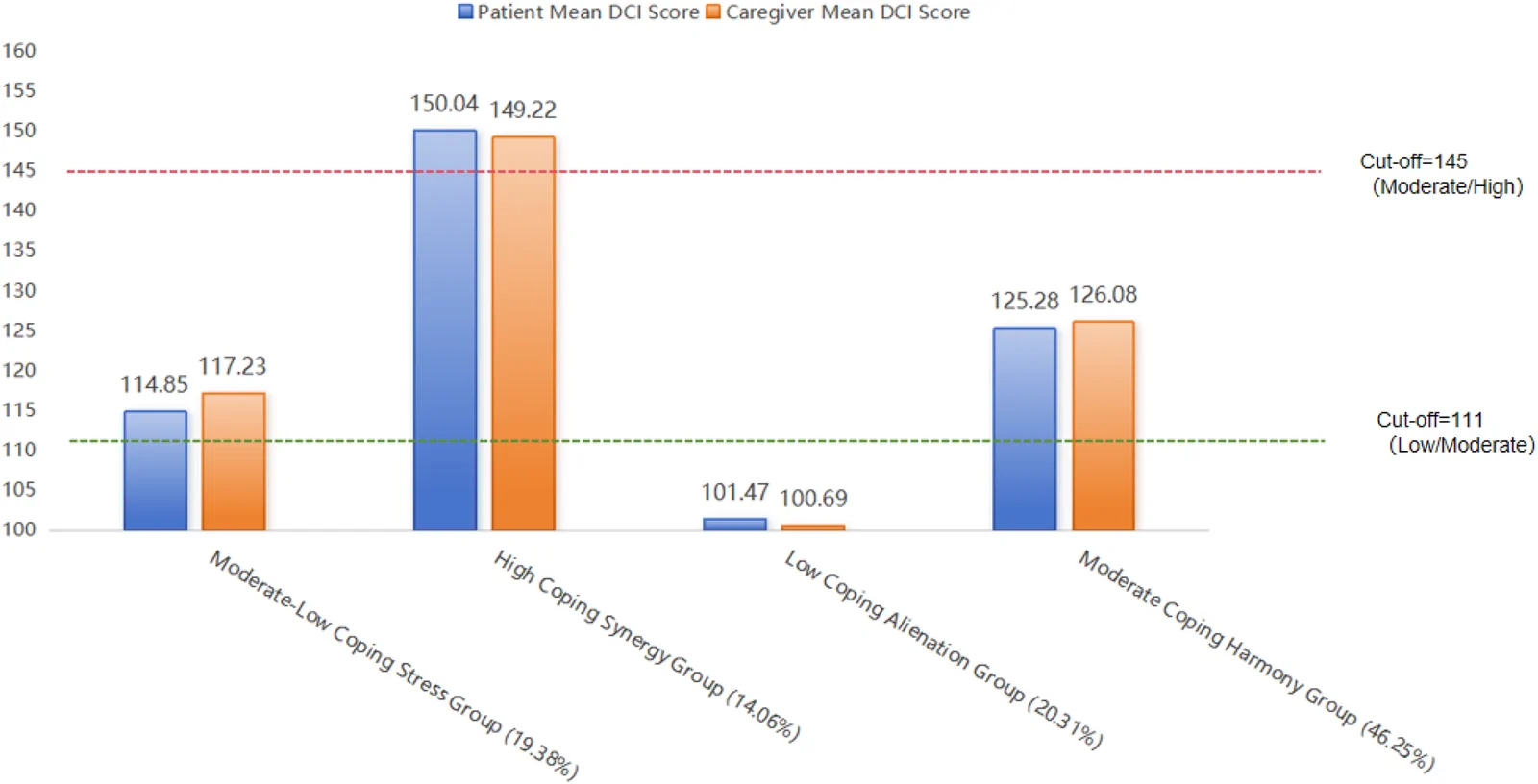
Potential Profile Analysis Diagram of Dyadic Coping for Patients and Their Primary Caregivers. The percentages in parentheses below each group label indicate the proportion of the corresponding latent trait in the total sample. The x-axis shows full standardized profile names, and the y-axis represents total DCI scores (35–175), with dashed cut-off lines at 111 and 145 separating low, moderate and high coping levels.

Based on this, latent profiles of dyadic coping for patients and their primary caregivers were constructed. The characteristics of each latent category within the dyadic coping pattern 4 for patients and their primary caregivers were analyzed and labeled. Category 1: 62 patient-primary caregiver pairs (19.38%). Their dyadic coping scores were (114.85 ± 4.47) and (117.23 ± 4.82), respectively—below the overall mean but higher than Category 3. Thus named Moderate-Low Coping Stress Group. Category 2: 45 pairs of patients and primary caregivers (14.06%). Their dyadic coping scores were (150.04 ± 6.83) and (149.22 ± 8.59), respectively, indicating higher levels compared to the overall average. Thus named High Coping Synergy Group; Category 3 patients and primary caregivers numbered 65 pairs (20.31%). Their dyadic coping scores were (101.47 ± 6.54) and (100.69 ± 7.54), respectively, which were lower than the overall average. This group was thus named the Low Coping Alienation Group. Category 4: 148 pairs of patients and primary caregivers (46.25%). Their dyadic coping scores were (125.28 ± 16.77) and (126.08 ± 17.13), respectively. These scores were above the overall average but below Category 2, thus named Moderate Coping Harmony Group (FIGURE 2).

### 3.3 Univariate analysis of factors influencing dyadic coping latent profiles in heart failure patients and their primary caregivers

Univariate analyses were conducted using the dyadic coping latent profiles of heart failure patients and their primary caregivers as the dependent variable, with general demographic questionnaires and self-rated fatigue scores as independent variables. Results indicated that the patient's marital status, per capita monthly household income, NYHA class, self-regulatory fatigue score, and the primary caregiver's gender, BMI, educational level, duration of caregiving, confidence in caregiving, communication with the patient, understanding of the patient's condition, and self-regulatory fatigue score were statistically significant (*P* < 0.05) when comparing different profiles of dyadic coping among patients and primary caregivers (Table 4).

**Table 4:** Univariate analysis of factors associated with latent profiles of dyadic coping in patients and their primary caregivers [*x¯±s/n(%)*].

Characteristics	Item	Moderate-low coping stress group (*n* = 62)	High coping synergy group (*n* = 45)	Low coping alienation group (*n* = 65)	Moderate coping harmony group (*n* = 148)	Statistic	*P*
Patients	Marital Status					21.367^a^	0.011*
	Married	48 (77.40)	42 (93.30)	55 (84.60)	126 (85.10)		
	Divorced	4 (6.50)	0 (0.00)	2 (3.10)	6 (1.90)		
	Unmarried	0 (0.00)	1 (2.20)	0 (0.00)	5 (1.60)		
	Widowed	10 (16.10)	2 (4.40)	8 (12.30)	38 (11.90)		
	Monthly Per Capita Household Income (CNY)					44.468^a^	<0.001**
	<3,000	13 (21.00)	3 (6.70)	25 (38.50)	29 (19.60)		
	3,000–5,000	30 (48.40)	16 (35.60)	36 (55.40)	82 (55.40)		
	>5,000	19 (30.60)	26 (57.80)	4 (6.20)	37 (25.00)		
	NYHA					45.804^a^	<0.001**
	II	26 (41.90)	26 (57.80)	11 (16.90)	41 (27.70)		
	Ⅲ	24 (38.70)	18 (40.00)	31 (47.70)	91 (61.50)		
	IV	12 (19.40)	1 (2.20)	23 (35.40)	16 (10.80)		
	Self-Regulatory Fatigue	41.81 ± 4.83	36.89 ± 5.99	45.51 ± 4.45	40.07 ± 4.57	33.230^b^	<0.001 **
Caregiver	Gender					11.739^a^	0.008**
	Male	34 (54.80)	14 (31.10)	27 (41.50)	46 (31.10)		
	Female	28 (45.20)	31 (68.90)	38 (58.50)	102 (68.90)		
	BMI (kg/m^2^)	24.52 ± 3.15	23.30 ± 2.14	23.37 ± 1.91	23.41 ± 2.26	4.356^b^	0.005**
	Level of education					17.901^a^	0.036**
	Elementary school and below	18 (29.00)	18 (40.00)	20 (30.80)	38 (25.70)		
	Junior High	21 (33.90)	12 (26.70)	27 (41.50)	40 (27.00)		
	High School or Vocational School	9 (14.50)	3 (6.70)	12 (18.50)	36 (24.30)		
	College degree or higher	14 (22.60)	12 (26.70)	6 (9.20)	34 (23.00)		
	Years of Care (years)					31.886^a^	<0.001**
	0–2	51 (82.30)	38 (84.40)	31 (47.70)	105 (70.90)		
	3–5	9 (14.50)	7 (15.60)	24 (36.90)	34 (23.00)		
	6–8	0 (0.00)	0 (0.00)	6 (9.20)	5 (3.40)		
	≥9	2 (3.20)	0 (0.00)	4 (2.70)	4 (2.70)		
	Confidence in caring for patients					125.030^a^	<0.001**
	Yes	45 (72.60)	42 (93.30)	15 (23.10)	137 (92.60)		
	No	17 (27.40)	3 (6.70)	50 (76.90)	11 (7.40)		
	Communication with patients					248.636^a^	<0.001**
	Frequently	11 (17.70)	42 (93.33)	2 (3.10)	145 (98.00)		
	Occasionally	51 (82.30)	3 (6.67)	63 (96.90)	3 (2.00)		
	Understanding of the patient's condition					45.618^a^	<0.001**
	Unfamiliar	1 (1.60)	0 (0.00)	2 (3.10)	0 (0.00)		
	Somewhat familiar	48 (77.40)	21 (46.70)	60 (92.30)	86 (58.10)		
	Know a lot	13 (21.00)	24 (53.30)	3 (4.60)	62 (41.90)		
	Self-Regulatory Fatigue	38.39 ± 5.19	36.93 ± 8.91	42.71 ± 5.24	37.91 ± 4.07	14.440^b^	<0.001**

NYHA, New York Heart Association functional classification; BMI, Body Mass Index; a, Chi-square test; b, One-way analysis of variance; *, *P* < 0.05; **, *P* < 0.01.

### 3.4 Multivariate analysis of influencing factors for latent profiles of dyadic coping in patients with chronic heart failure and primary caregivers

Using the four dyadic latent profiles for heart failure patients and their primary caregivers identified by latent profile analysis as the dependent variable, with the moderate coping harmony group as the reference category, regression models were constructed with variables showing statistical significance in univariate analysis as independent variables. The parallelism *test* yielded *χ^2^*= 253.359, *P* < 0.001, failing to meet the condition (*P* > 0.05). Therefore, unordered multinomial logistic regression analysis was employed to explore independent factors influencing each latent category of dyadic coping among heart failure patients and their primary caregivers.

Results from the multinomial logistic regression analysis indicate that, compared to moderate coping harmony group, patient self-regulatory fatigue and communication between primary caregivers and patients are predictive factors for the Moderate-Low Coping Stress Group. Patient self-regulatory fatigue, per capita monthly household income, New York Heart Association functional class, and primary caregiver education level were predictive factors for High Coping Synergy Group; Patient self-regulatory fatigue, per capita monthly household income, and communication between primary caregivers and patients were predictive factors for Low Coping Alienation Group (Table 5).

**Table 5:** Multivariate analysis of dyadic coping latent profiles for patients and primary caregivers.

Category	Variable	*B*	*OR*	*95% CI*	*P*
Moderate-Low Coping Stress Group	Patient Self-Regulatory Fatigue	0.175	1.192	1.037–1.370	0.014*
	Communication with Patients - Occasionally	6.090	441.482	72.229–2,698.449	<0.001**
High Coping Synergy Group	Patient Self-Regulatory Fatigue	−0.111	0.895	0.955–1.133	0.018*
	Monthly household income per capita < 3,000 yuan	−1.951	0.142	0.029–0.699	0.016*
	Per capita monthly household income 3,000–5,000 yuan	−1.664	0.189	0.070–0.511	<0.001**
	NYHA Class II	2.570	13.067	1.300–131.355	0.029*
	Educational attainment: Elementary school and below	1.623	5.071	1.440–17.850	0.011*
Low Coping Alienation Group	Patient Self-Regulatory Fatigue	0.244	1.276	1.071–1.520	0.006**
	Monthly household income per capita < 3,000 yuan	2.912	18.392	1.269–266.655	0.033*
	Per capita monthly household income 3,000–5,000 yuan	2.344	10.420	1.059–102.528	0.045*
	Communication with patients Occasionally	8.059	3,162.567	246.536–40,569.457	<0.001**

NYHA, New York Heart Association functional classification; *, *P* < 0.05; **, *P* < 0.01.

## 4 Discussion

### 4.1 Dyadic coping levels in patients with chronic heart failure and their primary caregivers

This study found that the total dyadic coping scores for heart failure patients and primary caregivers were (125.28 ± 16.77) and (126.08 ± 17.13), respectively, both indicating moderate levels. As the core mechanism for joint disease management between patients and primary caregivers, moderate dyadic coping is correlated with collaborative interactions formed during long-term care. This allows them to manage the disease to a certain extent together, while also reflecting that their coping abilities are correlated with multiple variables and have room for improvement. From the patient's perspective, recurrent chronic heart failure episodes are linked to persistent symptoms including dyspnea, fatigue, and edema. Such symptoms correlate with impaired quality of life and restricted social engagement, which is associated with difficulty sustaining regular social roles and relationships. Beyond daily care and disease management tasks, primary caregivers also face illness-related financial stress, which corresponds with varying degrees of disrupted daily routines (23). The combined stressors experienced by both parties are associated with restrained dyadic coping capacity.Higher levels of positive dyadic coping correlate with better quality of life, alleviate the caregiving burden on primary caregivers, and enhance the mental health of both parties. Clinical healthcare providers should prioritize patients and primary caregivers with low dyadic coping levels, which may correlate with stronger collaborative coping efficacy and better overall health outcomes of the two groups.

### 4.2 Analysis of potential category characteristics of dyadic coping in chronic heart failure patients and their primary caregivers

Through latent profile analysis, this study identified four dyadic coping patterns among patients with chronic heart failure and their primary caregivers: High Coping Synergy Group, Low Coping Alienation Group, Moderate Coping Harmony Group, and Moderate-Low Coping Stress Group. Wang Bingbing conducted latent profile analysis on the dyadic coping of middle-aged and young stroke patients and their spouses (24), categorizing their dyadic coping into three groups: low-dyadic-coping couples, moderate-coping couples, and high-coping couples. In this study, the High Coping Synergy Group is correlated with a high level of dyadic coping ability, which is accompanied by joint execution of disease management plans, active utilization of medical resources, and timely mutual emotional perception and support between dyad members. The Low Coping Alienation Group is associated with insufficient coping strategies, experiencing communication deficits in disease management, suppressed emotional expression, and widespread symptoms of self-regulatory fatigue. This profile indicates the group corresponds to a vicious cycle of “resource depletion-ineffective coping-escalating stress” (25). Such status is correlated with psychological exhaustion from prolonged disease burden and insufficient training in effective coping skills (26). The Moderate Coping Harmony Group shows medium dyadic coping scores, with consistent interactive strategies, fundamental communication competence, and mutual willingness to address illness-related problems; this subgroup accounts for the largest proportion of dyads in this research. For the Moderate-Low Coping Stress Group, both parties exhibited coping levels slightly below average. While they maintained basic disease-related communication, they lacked deep collaboration and emotional resonance. Fragmented, individual-oriented coping behaviors in this subgroup are correlated with a higher probability of tense patient-caregiver interactions caused by communication barriers.

### 4.3 Analysis of key influencing factors and mechanisms in the dyadic coping of chronic heart failure patients and their primary caregivers based on latent profile analysis

#### 4.3.1 Communication frequency

The study found that communication between primary caregivers and patients is strongly correlated with dyadic coping patterns. Analysis indicates that compared to “frequent” communication, “occasional” communication is a significant correlational indicator associated with membership the Moderate-Low Coping Stress Group (*OR* = 441.482) and Low Coping Alienation Group (*OR* = 3,162.567).This aligns with findings from Roth (27), which reported that frequent positive communication correlates with elevated dyadic coping scores. In the moderate-to-low coping stress group, mismatched coping resources are associated with poor communication, where one party seeks connection while the other adopts avoidance strategies, a feature that co-occurs with weaker dyadic coping capacity, matching the conclusions of Eldridge (28).In Low Coping Alienation Group, scarce emotional communication and limited mutual support are accompanied by mutual emotional withdrawal. Otto pointed out that low communication proficiency is associated with a higher care burden (29). From a clinical perspective, assessing and promoting communication between patients and primary caregivers should be central to interventions. clinical staff may prioritize communication training and rebuild channels for emotional and disease-related information exchange to support better dyadic interaction.

#### 4.3.2 Self-regulatory fatigue

This study found that patients' self-regulatory fatigue is a significant correlational indicator linked to membership in the Moderate-Low Coping Stress Group (*OR* = 1.192) and Low Coping Alienation Group (*OR* = 1.276). This cross-sectional association reflects that avoidance and ineffective coping strategies co-occur alongside continuous consumption of psychological resources without adequate replenishment. Elevated self-regulatory fatigue correlates with a higher likelihood of unfavorable psychosocial outcomes (30). Greater self-regulatory fatigue is associated with a tendency to adopt negative coping styles among patients and primary caregivers. For dyads in the Low Coping Alienation Group with dual depletion of psychological resources, elevated self-regulatory fatigue coexists with a lack of capacity to offer or receive supportive interactions, alongside mutual emotional withdrawal. Research indicates that heart failure patients face numerous drains on their psychological resources, such as knowledge gaps, diagnostic and treatment uncertainties, anxiety, depression, decision-making pressures, and complex self-management tasks.Such stressors are correlated with rapid depletion of psychological reserves and psychological strain (31).From a clinical perspective, medical staff may integrate assessments of psychological resource status into routine heart failure care, as self-regulatory fatigue is a correlate of impaired dyadic coping function. Joint fatigue management for patients and caregivers may correlate with sustained psychological resource reserves. Such practice is linked to improved individual well-being and stable supportive patient-caregiver dyadic bonds.

#### 4.3.3 New York Heart Association functional class

This study found that NYHA Stage II status is significantly correlated with higher odds of belonging to the High Coping Synergy Group (*OR* = 13.067). Patients at this stage exhibit pronounced heart failure symptoms, which co-occur with increased disease awareness and enhanced coping efforts within dyads.Patients at this stage retain the capacity for daily self-care, and the primary caregiver's burden remains within their routine coping capabilities. Such manageable care pressure is accompanied by collaborative dyadic interactions rather than overwhelming strain, a feature previously documented in dyadic coping research (19). Research indicates that moderate caregiving stress correlates with stronger patient-caregiver cooperation via dyadic interaction processes (19). These findings resonate with the core concept of the “window of opportunity” in heart failure home care proposed by Kitko (32), which reports moderate disease challenges are linked to mobilization of family support resources.This cross-sectional association implies mild disease-related threats coexist with strong family cohesion. Targeted professional guidance delivered during this phase may correlate with favorable dyadic outcomes, though longitudinal research is required to verify directional effects.

#### 4.3.4 Socioeconomic status of the household

The associations between household economic indicators and dyadic coping are multifaceted. This study found that monthly per capita household income below 3,000 yuan acts as a significant negative correlational marker linked to membership in the High Coping Synergy Group, and a strong correlational indicator associated with classification into the Low Coping Alienation Group (*OR* = 18.392). Chronic heart diseases usually requires long-term medical expenditure, and financial outlays for disease management are correlated with patients' physical status. Household economic status is associated with how patient-caregiver dyads cope with treatment-related financial stress, consistent with the family stress model (33).Within this theoretical framework, financial strain correlates with impaired family function, difficult disease adjustment, elevated negative emotions, and more frequent family conflicts, rather than exerting direct causal effects. In addition, lower household income coexists with heavier caregiving load, which is further correlated with weakened joint disease management and poorer quality of life for both members of the dyad (5). Existing studies have reported that online health resources correlate with better treatment access and quality of life among low- and middle-income patients (34). Based on the above, healthcare providers should proactively assess household financial burdens when delivering basic medical guidance. By leveraging internet-based support systems tailored to patients’ and primary caregivers' circumstances, they can offer convenient, personalized medical consultations, remote condition monitoring, and health education services for chronic heart failure patients and their caregivers. Such service models may correlate with narrower financial-related gaps in disease management and fewer relational tensions linked to economic stress, though longitudinal trials are needed to confirm directional effects.

#### 4.3.5 Educational attainment

This study found that primary caregivers with educational attainment of elementary school or below were significantly associated with membership in the High Coping Synergy Group (OR = 5.071) after adjusting for patient self-regulatory fatigue, monthly household income per capita, and NYHA functional class. This finding is inconsistent with the results reported by Wang et al. (24), whose research on stroke patient-spouse dyads indicated that lower educational levels predicted weaker dyadic coping capacity. The inconsistency between the two studies may be attributed to differences in disease types, participant family composition and sample source. In this study, caregivers with low educational levels tended to rely on intimate spousal and intergenerational emotional support to jointly manage heart failure-related stress, which facilitated positive interactive behaviors and high levels of dyadic coping. From a clinical perspective, medical staff should avoid simply judging dyadic coping potential only according to primary caregivers' educational background when designing personalized intervention schemes. For dyads with lower educational attainment, clinical interventions can focus on activating internal family emotional connections to sustain stable collaborative coping interactions.

## 5 Conclusion

This study examined chronic heart failure patients and their primary caregivers as integrated units, identifying four heterogeneous dyadic coping patterns through latent profile analysis. This provides scientific grounds for developing personalized care interventions. Healthcare providers should formulate targeted interventions tailored to the category characteristics and factors correlated with each latent profile, aiming to boost dyadic coping levels of both parties.This study was limited to heart failure patients and their primary caregivers from a single hospital in Jiangsu Province, employing convenience sampling, which may introduce selection bias.The sample cannot represent heart failure dyads from primary care, community facilities or other regions. Multi-center studies across different medical tiers are required to verify our findings. Second, this cross-sectional study merely captures variable correlations and cannot confirm causal links. Longitudinal follow-up is needed to track dynamic shifts in dyadic coping patterns, and future multi-site longitudinal research will establish more credible dyadic intervention schemes.

## Data Availability

The raw data supporting the conclusions of this article will be made available by the authors, without undue reservation.
